# Supplementary material for: The Use of Intravenous Fosfomycin in Clinical Practice: A 5-Year Retrospective Study in a Tertiary Hospital in Italy
Source: Antibiotics (Basel). 2023 May 27;12(6):971. doi: 10.3390/antibiotics12060971 (PMC10295113; doi:10.3390/antibiotics12060971)
Supplement: Supplementary file 1 [file antibiotics-12-00971-s001.zip › antibiotics-2372427-supplementary.pdf]

antibiotics

MDPI

Article

The Use of Intravenous Fosfomycin in Clinical Practice: A 5-Year Retrospective Study in a Tertiary Hospital in Italy

Antonio Anastasia [1,2], Silvia Bonura [2,3], Raffaella Rubino [2,3], Giovanni Maurizio Giammanco [1,3,4], Irene Micciché [3,5], Maria Rita Di Pace [1], Claudia Colomba [1] and Antonio Cascio [1,2,3,*]

1 Department of Health Promotion, Mother and Child Care, Internal Medicine and Medical Specialties "G D'Alessandro", University of Palermo, 90127 Palermo, Italy; antonioanastasia90@gmail.com (A.A.); giovanni.giammanco@unipa.it (G.M.G.); mariarita.dipace@unipa.it (M.R.D.P.); claudia.colomba@unipa.it (C.C.)

2 Infectious and Tropical Disease Unit and Sicilian Regional Reference Center for the Fight against AIDS, AOU Policlinico "P. Giaccone", 90127 Palermo, Italy; silvia.bonura@policlinico.pa.it (S.B.); raffaella.rubino@policlinico.pa.it (R.R.)

3 Antimicrobial Stewardship Team, AOU Policlinico "P. Giaccone", Palermo, Italy; irene.micciche@policlinico.pa.it

4 Microbiology and Virology Unit, AOU Policlinico "P. Giaccone", 90127 Palermo, Italy

5 UOC Farmacia, AOU Policlinico "P. Giaccone", 90127 Palermo, Italy

* Correspondence: antonio.cascio03@unipa.it; Tel.: +39-091-23890632

Supplementary Materials

Table S1. Microbiological isolates (data from mono- and polybacterial infections).

Pathogen	*N* (%)
Klebsiella pneumoniae	193 (56.2)
Staphylococcus aureus	16 (4.6)
Staphylococcus epidermidis	5 (1.5)
Staphylococcus haemolyticus	1 (0.3)
Staphylococcus capitis	2 (0.6)
Staphylococcus hominis	1 (0.3)
Pseudomonas aeruginosa	42 (12.2)
Acinetobacter baumannii	36 (10.5)
Escherichia coli	24 (7)
Enterococcus faecalis	14 (4.1)
Enterococcus faecium	14 (4.1)
Enterobacter cloacae	8 (2.3)
Proteus mirabilis	7 (2)
Proteus vulgaris	3 (0.9)
Citrobacter freundii	3 (0.9)
Corynebacterium striatum	3 (0.9)
Serratia marcescens	2 (0.6)
Enterobacter aerogenes	1 (0.3)
Streptococcus gordonii	1 (0.3)

Table S2. Fosfomycin combination partners and number of cases.

Combination partner	N (%)
Cephalosporins	
▪ Ceftazidime/avibactam	122 (35.5)
▪ Cefiderocol	7 (2)
▪ Cefepime	2 (0.5)

▪ Ceftobiprole	4 (1.2)
▪ Ceftaroline	1 (0.3)
▪ Cefazolin	5 (1.5)
▪ Ceftolozane/tazobactam	1 (0.3)
▪ Ceftriaxone	2 (0.5)
Carbapenems	
▪ Meropenem	57 (16.6)
▪ Meropenem/vaborbactam	7 (2)
▪ Ertapenem	3 (0.9)
▪ Imipenem-cilastatin	2 (0.5)
Glycopeptides	
▪ Vancomycin	28 (8.1)
▪ Teicoplanin	7 (2)
Penicillins	
▪ Ampicillin	1 (0.3)
▪ Piperacillin/tazobactam	12 (3.5)
▪ Ampicillin/sulbactam	3 (0.9)
▪ Oxacillin	1 (0.3)
Tigecycline	8 (2.3)
Colistin	49 (14.2)
Daptomycin	39 (11.4)
Linezolid	22 (6.4)
Metronidazole	6 (1.7)
Aminoglycoside	7 (2)
Fluoroquinolones	6 (1.7)
Trimethoprim/sulfamethoxazole	11 (3.2)
Clindamycin	1 (0.3)
Doxycycline	3 (0.9)
Rifampicin	1 (0.3)

Disclaimer/Publisher's Note: The statements, opinions and data contained in all publications are solely those of the individual author(s) and contributor(s) and not of MDPI and/or the editor(s). MDPI and/or the editor(s) disclaim responsibility for any injury to people or property resulting from any ideas, methods, instructions or products referred to in the content.
